# Nutrition knowledge and healthy-eating attitudes: the role of physical activity and lifestyle factors in adults

**DOI:** 10.3389/fnut.2026.1754963

**Published:** 2026-02-09

**Authors:** Bekir Erhan Orhan, Walaa Jumah Alkasasbeh, Aydın Karaçam, Umut Canlı, Niyazi Sıdkı Adıgüzel, Adam Tawfiq Amawi

**Affiliations:** 1 Faculty of Sports Sciences, Istanbul Aydın University, Istanbul, Türkiye; 2 Department of Physical Education, Faculty of Sports Science, The University of Jordan, Amman, Jordan; 3 Faculty of Sports Sciences, Bandırma Onyedi Eylül University, Balıkesir, Türkiye; 4 Faculty of Sports Sciences, Tekirdağ Namık Kemal University, Tekirdağ, Türkiye; 5 Department of Movement Sciences and Sports Training, School of Sports Sciences, The University of Jordan, Amman, Jordan

**Keywords:** behavioral clustering, healthy-eating attitudes, nutrition knowledge, physical activity, smoking

## Abstract

**Background:**

Diet quality and physical activity shape chronic-disease risk, yet the alignment between what adults know about nutrition and how they evaluate healthy eating remains unclear.

**Methods:**

Adults in Türkiye (*N* = 408; 46.3% women, 53.7% men; mean age 28.6 years) completed validated measures of nutrition knowledge (NKS) and attitudes toward healthy nutrition [ASHN; subscales: Information on Nutrition (IN), Emotion for Nutrition (EN), Positive Nutrition (PN), Malnutrition (MP)]. Lifestyle factors included smoking, alcohol use, and 7-day physical activity (sedentary/low/moderate/high). Analyses comprised t-tests, one-way ANOVA with Tukey tests, Pearson correlations, and multiple linear regression.

**Results:**

Knowledge and attitudes were only weakly aligned: higher NKS related to lower ASHN Total (*r* = −0.18, *p* < 0.001) and to IN (*r* = −0.21), PN (*r* = −0.11), and MP (*r* = −0.17); EN was null (*r* = −0.01). In regression, IN (*β* = −0.17, *p* = 0.001) and MP (*β* = −0.16, *p* = 0.006) were independently associated with lower NKS; overall fit was small (*R*^2^ = 0.063; *F*(4,403) = 6.815, *p* < 0.001). Attitudes varied strongly by physical activity High > Low/Moderate/Sedentary for ASHN Total (*F*(3,404) = 10.10, *η*^2^ = 0.07), IN (*F* = 8.10), and PN (*F* = 11.11); EN showed no group difference. Smoking showed a paradox: knowledge was higher among heavier smokers (*F*(3,404) = 3.47, *p* = 0.010), whereas attitudes were less favorable (Never/Former > ≥ 11/day for ASHN Total; *F* = 6.47, *η*^2^ = 0.04). Non-drinkers reported higher ASHN than drinkers (*t*(406) = 2.48, *p* = 0.013) with lower MP (*t*(406) = 3.65, *p* < 0.001). Education displayed clear stepwise gradients for attitudes (e.g., ASHN Total *F* = 18.97, *η*^2^ = 0.12), but not for knowledge. Age correlated positively with ASHN Total (*r* = 0.27) and EN/PN/MP, but not with NKS (*r* = −0.07).

**Conclusion:**

In this adult sample, nutrition knowledge and healthy-eating attitudes were largely distinct. Attitudes especially belief- and practice-oriented facets tracked physical activity and other lifestyle factors, whereas knowledge did not. Interventions should pair education with motivation, self-regulation, and contextual supports to convert knowledge into healthier eating.

## Introduction

Diet quality remains a central determinant of noncommunicable disease risk, operating alongside physical activity and other lifestyle practices across the life course (1–3). Contemporary public guidance emphasizes patterns rich in minimally processed foods, fruits and vegetables, whole grains, and appropriate energy balance, combined with regular moderate-to-vigorous activity (4). Aligning everyday eating with these recommendations is challenging because dietary choices are influenced by a combination of factors, including knowledge, values, motivation, self-regulation, habits, social context, and environmental constraints that affect time and cost (5–7).

National surveillance indicates that Türkiye faces a substantial burden of modifiable noncommunicable disease risk factors, providing a clear rationale for examining nutrition-related constructs in this context (8, 9). National monitoring data indicate that insufficient physical activity is common among adults and that excess body weight remains a widespread public-health concern (8). Official health statistics likewise show a continuing high prevalence of obesity among individuals aged 15 years and older (9). Consistent with these national indicators, recent evidence in Turkish adults suggests that nutrition-related attitudes vary across physical-activity and body-mass profiles, supporting the inclusion of lifestyle factors when examining evaluative nutrition constructs in Türkiye (10).

Health literacy and life-course perspectives underscore that capacities relevant to navigating food environments evolve with age, education, and accumulated experience, and they extend well beyond the possession of factual information (11, 12). Evidence across diverse adult populations shows that higher factual understanding is only inconsistently mirrored by healthier appraisals or intentions, pointing to gaps between what people know and how they evaluate or plan to act (13, 14). These gaps matter for intervention design because programs that privilege information alone may leave the motivational and regulatory ingredients of behavior change unaddressed.

Dual-process accounts and related social-cognition models position attitudes, perceived control, and habit strength as proximal to behavior, whereas declarative knowledge exerts influence chiefly when it is embedded in motivated, self-regulated action (15, 16). Meta-analytic syntheses consistently find that behavior-change techniques targeting self-regulatory processes goal setting, action and coping planning, self-monitoring, and feedback yield stronger effects than education alone, particularly when delivered in autonomy-supportive ways (17, 18). Randomized and digitally mediated interventions that incorporate motivational interviewing or self-determination theory–informed components further demonstrate added value beyond purely informational content (19–22).

Large population datasets reveal clustering of physical activity, smoking, alcohol use, and dietary behaviors within coherent profiles that appear to share determinants such as self-efficacy, health identity, and planning skill (23–27). Within this constellation, public-health anchors for weekly activity approximately 150 min of moderate intensity offer pragmatic thresholds for classifying exposure (4). Because attitudes index motivational readiness and regulatory capacity, they should align more closely with other health-promoting practices than factual knowledge alone (28).

Cohabitation and social ties can facilitate meal planning and reinforce norms that support healthier eating, whereas living alone is often associated with less favorable dietary patterns (29, 30). These sociodemographic gradients may strengthen evaluative orientations toward healthy eating even when factual knowledge has already reached a basic threshold through schooling or media exposure (11, 31, 32). Together, this literature suggests that attitudes may track lifestyle and social positioning more reliably than knowledge, with implications for the targeting of limited intervention resources.

This study (i) profiles adults’ nutrition knowledge and healthy-eating attitudes; (ii) quantifies their correspondence and any knowledge–attitude gap; and (iii) tests for differences across lifestyle factors physical activity, smoking, alcohol use, and family history of chronic illness as well as sociodemographic characteristics (education, age, gender, marital status). Guided by behavioral-clustering theory, the *a priori* expectation is that attitudes being closer to motivation and self-regulation will exhibit stronger and more consistent gradients across lifestyle factors than knowledge, whereas knowledge will show weaker or no gradients after accounting for sociodemographics. A further objective is to evaluate whether healthy-eating attitudes are independently associated with nutrition knowledge in multivariable models, anticipating only small associations if the constructs operate through distinct pathways.

The aim of the study is to determine the relationship between adults’ nutrition knowledge and their attitudes toward healthy eating and to assess how both vary by lifestyle and sociodemographic characteristics, thereby informing whether future programs should target informational, motivational, or contextual strategies for maximal impact.

## Methods

### Model

This descriptive, cross-sectional correlational study examined associations between adults’ nutrition knowledge and attitudes toward healthy nutrition and compared scores across key sociodemographic characteristics and lifestyle factors. Because data were collected at a single time point, findings should be interpreted as associative and do not permit causal inference (33).

### Study group

The study group was assembled through convenience sampling, a pragmatic approach commonly used when rapid recruitment is needed (34). Eligible individuals were ≥18 years, residing in Türkiye, able to provide informed consent, and able to complete an online self-report form. The final analytic sample comprised 408 adults (46.3% women, 53.7% men; M_age = 28.60 years). Descriptive characteristics of the sample are presented in Table 1.

**Table 1:** Normality test results (skewness and kurtosis).

Variable	Skewness	SE	Kurtosis	SE
NKS Total	0.38	0.12	−0.42	0.24
ASHN Total	−0.39	0.12	−0.15	0.24
IN	−1.09	0.12	1.37	0.24
EN	−0.19	0.12	−0.54	0.24
PN	−0.26	0.12	−0.45	0.24
MP	−0.71	0.12	−0.07	0.24

SE, standard error; NKS, Nutrition Knowledge Scale; ASHN, Attitude Scale for Healthy Nutrition; IN, Information on Nutrition; EN, Emotion for Nutrition; PN, Positive Nutrition; MP, Malnutrition.

### Sample size and power

A total of 490 adults initiated the questionnaire; after exclusions for ineligibility or incomplete key data, the analytic sample was N = 408. Although sampling was by convenience, this N affords narrow confidence intervals and adequate sensitivity for the planned analyses. For example, the 95% CI half-width for group means is approximately ±1.2 points for ASHN (SD ≈ 12) and ±2.4 points for NKS (SD ≈ 24.8). For two-group comparisons with sizes similar to the gender split (*n*₁ = 189, *n*₂ = 219; *α* = 0.05, two-tailed), the study is sensitive to small effects of about *d* ≈ 0.28–0.30. One-way ANOVAs with four groups (e.g., physical-activity categories) have adequate sensitivity for small-to-moderate omnibus effects (≈ *η*^2^ ≥ 0.03–0.06). For correlations, effects of *r* ≈ 0.14 are detectable at ~80% sensitivity (*α* = 0.05) (35, 36). These values indicate the study is equipped to estimate means with reasonable precision and to detect small associations and small-to-moderate group differences typical of lifestyle and attitude research.

### Data collections

#### Nutrition knowledge scale (NKS)

Adapted into Turkish by Öngün Yılmaz et al. (37) for healthy adults, the NKS is a 31-item, 5-point Likert instrument that assesses general nutrition knowledge. Items are scored 4 → 0 from Strongly agree to Strongly disagree; misinformation items are reverse-coded so that higher totals reflect greater knowledge. Consistent with the Turkish validation, reverse-scored items are 1, 5, 6, 9, 10, 16, 17, 21, 27, 28 and the scale functions as a single-factor measure. The total score ranges 0–124. Psychometric properties reported by Yılmaz et al. (2021) support good reliability and construct validity: KMO = 0.862 with a significant Bartlett test (χ^2^ = 3869.244, *p* < 0.001); Cronbach’s *α* = 0.85 (internal consistency) and ICC = 0.86 (test–retest). Confirmatory factor analysis indicated acceptable–good fit (CMIN/df = 2.347, RMSEA ≈ 0.04–0.05, CFI ≈ 0.94, GFI ≈ 0.98–0.99, AGFI ≈ 0.97). For descriptive interpretation in that study, empirical cut-points were used (e.g., low ≤79, medium 80–90, high 91–100, very high ≥101) (37).

#### Attitude scale for healthy nutrition (ASHN)

The ASHN is a 21-item, 5-point Likert instrument developed by Tekkurşun Demir and Cicioğlu (38) to assess adults’ attitudes toward healthy eating. Items are rated from 1 = Strongly Disagree to 5 = Strongly Agree and summed to yield a total score from 21 to 105, with higher scores indicating more favorable attitudes. The scale comprises four subdomains using the English labels throughout this manuscript: Information on Nutrition (IN; items 1–5), Emotion for Nutrition (EN; items 6–11), Positive Nutrition (PN; items 12–16), and Malnutrition (MP; items 17–21). As in the original validation, negatively worded items (6–11, 17–21) are reverse-coded prior to scoring. For descriptive interpretation, we used published bands: 21–22 very low, 23–42 low, 43–63 moderate, 64–84 high, and 85–105 very high. The developers reported good psychometrics (internal consistency: *α* = 0.90 for IN, *α* = 0.84 for EN, α = 0.75 for PN, *α* = 0.83 for MP; CFA fit: *χ*^2^/df = 1.71, RMSEA = 0.04, PGFI = 0.74, PNFI = 0.82, GFI = 0.92, AGFI = 0.90, IFI = 0.98, NFI = 0.95, CFI = 0.98), supporting a four-factor structure with a higher-order attitude construct (38).

#### Sociodemographic and lifestyle factors

Participants reported age, gender, marital status, and highest educational attainment. Lifestyle factors were assessed as smoking status, alcohol use, and physical activity. In addition, family history of chronic illness was recorded as a health-related background characteristic (presence of at least one chronic condition in a first-degree relative). Physical activity was self-reported via a single 7-day frequency item in the online questionnaire: “During the past 7 days, on how many days did you perform ≥30 minutes of physical activity (e.g., walking, running, sport, or exercise)?” Because this measure relied on self-report, responses may be influenced by recall and social desirability. Responses were categorized as sedentary (0 days), low (1–2 days), moderate (3–4 days), and high (≥5 days/week), aligning with WHO guidance recommending at least 150 min/week of moderate-intensity physical activity (4).

### Data analysis

Data were screened for entry errors, outliers, distributional assumptions, and multicollinearity prior to analysis; no miscoded values were detected. Analyses were conducted in SPSS 27. Descriptive statistics were computed for all variables. Normality was evaluated using skewness and kurtosis (|skewness| and |kurtosis| ≤ 1.5) and visual inspection of histograms and Q–Q plots; normality indices for the study variables are summarized in Table 1 and indicated acceptable departures from normality for the planned parametric analyses (39). For two-group comparisons, independent-samples *t* tests were used, and for multi-group comparisons, one-way ANOVA with Tukey *post hoc* tests was applied. Bivariate associations were examined using Pearson product–moment correlations. To examine whether ASHN subdimensions were independently associated with nutrition knowledge, multiple linear regression was conducted with ASHN subdimensions—Information on Nutrition (IN), Emotion for Nutrition (EN), Positive Nutrition (PN), and Malnutrition (MP)—entered simultaneously as independent variables and the NKS total score as the dependent variable. Regression assumptions were evaluated using residual diagnostics: normality was assessed via histograms and Normal P–P/Q–Q plots (supported by the Shapiro–Wilk test), and linearity and homoscedasticity were examined using standardized residuals versus predicted values plots; no substantial deviations were observed. Multicollinearity was evaluated using tolerance and variance inflation factor (VIF) statistics (all VIF < 5). Statistical significance was set at *p* < 0.05. Effect sizes were reported alongside *p*-values (Cohen’s *d* for *t* tests, *η*^2^ for ANOVA, and Pearson’s *r* for correlations) and interpreted using conventional benchmarks (40).

### Ethical approval and informed consent

Approved by the Ethics Committee of the Faculty of Social and Human Sciences, Istanbul Aydın University (Decision No. 2025/7, June 25, 2025), and conducted in accordance with the Declaration of Helsinki. Participation was voluntary, and written informed consent was obtained after participants were informed of the study and their right to withdraw at any time. To protect privacy, no directly identifying data were collected; responses were pseudonymized, stored on password-protected systems, and accessible only to the research team. Data were analyzed and reported in aggregate to maintain confidentiality.

## Results

The sample included 408 adults (46.3% women, *n* = 189; 53.7% men, *n* = 219; mean age = 28.60 years). By highest educational attainment, 48.5% (*n* = 198) had completed high school, 17.2% (*n* = 70) held an associate degree, 24.0% (*n* = 98) held a bachelor’s degree, and 10.3% (*n* = 42) held a master’s degree. Regarding marital status, 65.2% (*n* = 266) were single and 34.8% (*n* = 142) were married (see Table 2).

**Table 2:** Sample characteristics (*N* = 408).

Variable	Category	*n*	%
Gender	Female	189	46.3
	Male	219	53.7
Marital status	Single	266	65.2
	Married	142	34.8
Highest education	High school	198	48.5
	Associate	70	17.2
	Bachelor’s	98	24.0
	Master’s	42	10.3

As shown in Table 3, men scored higher on NKS than women, *t*(406) = −3.70, *p* < 0.001, mean difference (Male − Female) = 8.98 [95% CI (4.21, 13.75)], *d* = 0.36. ASHN Total and its subscales did not differ by gender (e.g., mean difference (Male − Female) = −0.63, 95% CI [−3.01, 1.75] for ASHN Total; all *p* > 0.05).

**Table 3:** Independent samples *t*-test results for NKS and ASHN scores by gender (female *n* = 189; male *n* = 219).

Variables	Female 𝑋̄ (SD)	Male 𝑋̄ (SD)	*t*	df	*p*	Cohen’s d	Mean diff (95% CI)
NKS Total	44.23 (22.04)	53.21 (26.25)	−3.70	406	< 0.001	−0.36	+8.98 [4.21, 13.75]
ASHN Total	76.96 (12.01)	76.33 (12.31)	0.52	406	0.600	0.05	−0.63 [−3.01, 1.75]
IN	20.86 (3.87)	20.61 (4.33)	0.62	406	0.534	0.06	−0.25 [−1.04, 0.54]
EN	19.08 (4.96)	19.15 (4.54)	−0.14	406	0.888	−0.01	+0.07 [−0.91, 1.05]
PN	17.38 (4.40)	17.44 (4.10)	−0.14	406	0.888	−0.01	+0.06 [−0.78, 0.90]
MP	19.63 (4.03)	19.13 (4.49)	1.18	406	0.238	0.11	−0.50 [−1.33, 0.33]

Values are mean ± SD. NKS, Nutrition Knowledge Scale; ASHN, Attitude Scale for Healthy Nutrition; IN, Information on Nutrition; EN, Emotion for Nutrition; PN, Positive Nutrition; MP, Malnutrition. Mean diff = second group − first group; 95% CI, confidence interval for the mean difference. Two-tailed tests; *p* < 0.05 considered significant.

As shown in Table 4, married participants scored higher on ASHN than single participants, *t*(406) = 3.31, *p* < 0.001, mean difference (Single − Married) = −4.15 [95% CI (−6.61, −1.69)], *d* = 0.34. Subscales showed a similar pattern for EN, *t*(406) = 5.06, *p* < 0.001, mean difference (Single − Married) = −2.42 [95% CI (−3.36, −1.48)], *d* = 0.52; for PN, *t*(406) = 2.64, *p* = 0.009, mean difference (Single − Married) = −1.15 [95% CI (−2.01, −0.29)], *d* = 0.27; and for MP, *t*(406) = 3.06, *p* = 0.002, mean difference (Single − Married) = −1.35 [95% CI (−2.22, −0.48)], *d* = 0.31. IN did not differ by marital status, *t*(406) = −1.83, *p* = 0.068, mean difference (Single − Married) = 0.82 [95% CI (−0.06, 1.70)], *d* = −0.19. NKS Total did not differ, *t*(406) = 0.68, *p* = 0.490, mean difference (Single − Married) = −1.76 [95% CI (−6.85, 3.33)].

**Table 4:** Independent samples t-test results for NKS and ASHN scores by marital status (Married *n* = 142; Single *n* = 266).

Variables	Married 𝑋̄ (SD)	Single 𝑋̄ (SD)	*t*	df	*p*	Cohen’s d	Mean diff (95% CI)
NKS Total	50.20 (26.93)	48.44 (23.56)	0.68	406	0.490	0.07	−1.76 [−6.85, 3.33]
ASHN Total	79.33 (12.78)	75.18 (11.59)	3.31	406	< 0.001	0.34	−4.15 [−6.61, −1.69]
IN	20.21 (4.66)	21.03 (3.78)	−1.83	406	0.068	−0.19	+0.82 [−0.06, 1.70]
EN	20.69 (4.52)	18.27 (4.63)	5.06	406	< 0.001	0.52	−2.42 [−3.36, −1.48]
PN	18.16 (4.16)	17.01 (4.23)	2.64	406	0.009	0.27	−1.15 [−2.01, −0.29]
MP	20.24 (4.23)	18.89 (4.25)	3.06	406	0.002	0.31	−1.35 [−2.22, −0.48]

Values are mean ± SD. NKS, Nutrition Knowledge Scale; ASHN, Attitude Scale for Healthy Nutrition; IN, Information on Nutrition; EN, Emotion for Nutrition; PN, Positive Nutrition; MP, Malnutrition. Mean diff = second group − first group; 95% CI, confidence interval for the mean difference. Two-tailed tests; *p* < 0.05 considered significant.

As shown in Table 5, only IN differed by family chronic illness, *t*(406) = −2.41, *p* = 0.016, mean difference (No − Yes) = 1.01 [95% CI (0.19, 1.83)], *d* = 0.24. NKS Total, ASHN Total, EN, PN, and MP did not differ between groups [all *p* > 0.05; e.g., ASHN Total mean difference (No-Yes) = 1.11, 95% CI (−1.34, 3.56)].

**Table 5:** Independent samples *t*-test results for NKS and ASHN scores by family chronic illness (Yes *n* = 155; No *n* = 253).

Variables	Yes 𝑋̄ (SD)	No 𝑋̄ (SD)	*t*	df	*p*	Cohen’s d	Mean diff (95% CI)
NKS Total	50.39 (25.57)	48.24 (24.28)	0.85	406	0.390	0.08	−2.15 [−7.12, 2.82]
ASHN Total	75.94 (11.79)	77.05 (12.38)	−0.89	406	0.370	−0.09	+1.11 [−1.34, 3.56]
IN	20.10 (4.19)	21.11 (4.04)	−2.41	406	0.016	−0.24	+1.01 [0.19, 1.83]
EN	19.53 (4.48)	18.86 (4.87)	1.38	406	0.168	0.14	−0.67 [−1.62, 0.28]
PN	16.92 (3.85)	17.71 (4.44)	−1.83	406	0.067	−0.18	+0.79 [−0.06, 1.64]
MP	19.38 (4.24)	19.35 (4.32)	0.05	406	0.954	0.00	−0.03 [−1.21, 1.15]

Values are mean ± SD. NKS, Nutrition Knowledge Scale; ASHN, Attitude Scale for Healthy Nutrition; IN, Information on Nutrition; EN, Emotion for Nutrition; PN, Positive Nutrition; MP, Malnutrition. Mean diff = second group − first group; 95% CI, confidence interval for the mean difference. Two-tailed tests; *p* < 0.05 considered significant.

As shown in Table 6, ASHN was higher among non-users than users, *t*(406) = 2.48, *p* = 0.013, mean difference (Users − Non-users) = −3.13 [95% CI (−5.61, −0.65)], *d* = 0.26. MP was lower among non-users, *t*(406) = 3.65, *p* < 0.001, mean difference (Users − Non-users) = −1.61 [95% CI (−2.48, −0.74)], *d* = 0.38. NKS Total, IN, EN, and PN did not differ by alcohol use [all *p* > 0.05; e.g., NKS mean difference (Users − Non-users) = −1.30, 95% CI (−6.41, 3.81)].

**Table 6:** Independent samples *t*-test results for NKS and ASHN scores by alcohol use (Non-users *n* = 269; users *n* = 139).

Variables	Non-users 𝑋̄ (SD)	Users 𝑋̄ (SD)	*t*	df	*p*	Cohen’s d	Mean diff (95% CI)
NKS Total	49.50 (24.48)	48.20 (25.39)	0.50	406	0.610	0.05	−1.30 [−6.41, 3.81]
ASHN Total	77.69 (12.07)	74.56 (12.11)	2.48	406	0.013	0.26	−3.13 [−5.61, −0.65]
IN	20.84 (4.14)	20.51 (4.10)	0.74	406	0.459	0.07	−0.33 [−1.21, 0.55]
EN	19.35 (4.64)	18.66 (4.89)	1.40	406	0.162	0.14	−0.69 [−1.66, 0.28]
PN	17.58 (4.38)	17.07 (3.94)	1.14	406	0.255	0.12	−0.51 [−1.39, 0.37]
MP	19.91 (4.08)	18.30 (4.48)	3.65	406	< 0.001	0.38	−1.61 [−2.48, −0.74]

Values are mean ± SD. NKS, Nutrition Knowledge Scale; ASHN, Attitude Scale for Healthy Nutrition; IN, Information on Nutrition; EN, Emotion for Nutrition; PN, Positive Nutrition; MP, Malnutrition. Mean diff = second group − first group; 95% CI, confidence interval for the mean difference. Two-tailed tests; *p* < 0.05 considered significant.

As shown in Table 7, Educational attainment was not associated with NKS, *F*(3,404) = 0.52, *p* = 0.660, *η*^2^ < 0.01. In contrast, healthy-eating attitudes differed substantially by education, ASHN Total: *F*(3,404) = 18.97, *p* < 0.001, η^2^ = 0.12, with master’s degree reporting higher attitudes (*M* = 86.23, *SD* = 12.03) than high-school graduates (*M* = 72.84, *SD* = 10.81). The same pattern was observed for EN (*F*(3,404) = 19.26, *p* < 0.001, *η*^2^ = 0.12; MD *M* = 22.61 vs. HS *M* = 17.63) and for IN, PN, and MP (all *p* ≤ 0.006; *η*^2^ = 0.03–0.06), indicating an education gradient primarily for attitudinal domains rather than knowledge.

**Table 7:** One-way ANOVA results for NKS and ASHN (total and subscales) by highest educational attainment.

Variables	HS (*n* = 198) *M* ± SD	AS (*n* = 70) *M* ± SD	BA (*n* = 98) *M* ± SD	MD (*n* = 42) *M* ± SD	*F*(3,404)	*p*	η^2^	Tukey pairs (α = 0.05)
NKS Total	49.81 ± 22.32	45.98 ± 23.04	50.21 ± 26.01	47.90 ± 34.35	0.52	0.660	< 0.01	—
ASHN Total	72.84 ± 10.81	78.28 ± 12.22	78.96 ± 11.80	86.23 ± 12.03	18.97	< 0.001	0.12	MD > BA, AS, HS; BA > HS; AS > HS
IN	20.30 ± 4.37	20.74 ± 3.85	20.71 ± 3.99	22.76 ± 3.05	4.19	0.006	0.03	MD > BA, AS, HS
EN	17.63 ± 4.33	19.27 ± 4.90	20.51 ± 4.38	22.61 ± 4.29	19.26	< 0.001	0.12	MD > BA, AS, HS; BA > HS; AS > HS
PN	16.44 ± 4.20	18.20 ± 4.86	17.79 ± 3.30	19.76 ± 4.08	9.29	< 0.001	0.06	MD > BA, HS; BA > HS; AS > HS
MP	18.45 ± 4.18	20.07 ± 3.65	19.94 ± 4.53	21.09 ± 4.31	6.72	< 0.001	0.04	MD > HS; BA > HS; AS > HS

Values are mean ± SD. HS, High school; AS, Associate degree; BA, Bachelor’s degree; MD, Master’s degree. NKS, Nutrition Knowledge Scale; ASHN, Attitudes Toward Healthy Nutrition; IN, Information on Nutrition; EN, Emotion for Nutrition; PN, Positive Nutrition; MP, Malnutrition. Two-tailed tests; *p* < 0.05 considered significant.

As shown in Table 8, physical activity frequency showed no statistically significant differences in NKS (*F*(3,404) = 2.14, *p* = 0.090, *η*^2^ = 0.01). However, physical activity was associated with more favorable healthy-nutrition attitudes, ASHN Total: *F*(3,404) = 10.10, *p* < 0.001, *η*^2^ = 0.07, with the high-activity group scoring higher (*M* = 82.45, *SD* = 9.88) than the sedentary group (*M* = 71.22, *SD* = 13.20). Differences were also evident for IN (*F*(3,404) = 8.10, *p* < 0.001, η^2^ = 0.05) and PN (F(3,404) = 11.11, *p* < 0.001, *η*^2^ = 0.07), whereas EN did not differ across activity levels (*F*(3,404) = 1.74, *p* = 0.150). MP varied modestly (*F*(3,404) = 3.29, *p* = 0.020, *η*^2^ = 0.02), suggesting that activity frequency aligned more with information/positive nutrition orientations than with emotion-related attitudes.

**Table 8:** One-way ANOVA results for NKS and ASHN (total and subscales) by physical activity level (7-day frequency).

Variables	Sed (*n* = 54) *M* ± SD	Low (*n* = 140) *M* ± SD	Mod (*n* = 148) *M* ± SD	High (*n* = 66) *M* ± SD	*F*(3,404)	*p*	η^2^	Tukey pairs (*α* = 0.05)
NKS Total	41.31 ± 17.41	50.85 ± 26.64	50.28 ± 24.60	48.83 ± 25.45	2.14	0.090	0.01	—
ASHN Total	71.22 ± 13.20	75.16 ± 11.60	77.39 ± 12.15	82.45 ± 9.88	10.10	< 0.001	0.07	Low > Sed; Mod > Sed; High > Low, Mod, Sed
IN	19.25 ± 4.91	20.22 ± 3.84	20.90 ± 4.10	22.62 ± 3.35	8.10	< 0.001	0.05	Mod > Sed; High > Low, Mod, Sed
EN	18.53 ± 5.42	18.57 ± 4.55	19.57 ± 4.57	19.74 ± 4.78	1.74	0.150	0.01	—
PN	15.42 ± 4.26	17.08 ± 4.02	17.45 ± 4.46	19.65 ± 3.09	11.11	< 0.001	0.07	Low > Sed; Mod > Sed; High > Low, Mod, Sed
MP	18.00 ± 4.72	19.28 ± 3.85	19.45 ± 4.47	20.43 ± 4.15	3.29	0.020	0.02	Mod > Sed; High > Sed

Values are mean ± SD. Sed = Sedentary (0 days/week); Low = 1–2 days/week; Mod = 3–4 days/week; High = ≥5 days/week. NKS, Nutrition Knowledge Scale; ASHN, Attitudes Toward Healthy Nutrition; IN, Information on Nutrition; EN, Emotion for Nutrition; PN, Positive Nutrition; MP, Malnutrition. Two-tailed tests; *p* < 0.05 considered significant.

As shown in Table 9, smoking status was associated with small but significant differences in NKS (*F*(3,404) = 3.47, *p* = 0.010*, η*^2^ = 0.02), with heavier smokers reporting higher knowledge (≥11/day: *M* = 54.39, *SD* = 23.04) than former smokers (*M* = 41.73, *SD* = 22.44). In contrast, healthy-nutrition attitudes were less favorable among heavier smokers: ASHN Total: *F*(3,404) = 6.47, *p* < 0.001, *η*^2^ = 0.04, with never smokers (*M* = 78.53, *SD* = 10.86) and former smokers (M = 78.37, SD = 13.32) scoring higher than ≥11/day smokers (*M* = 72.30, *SD* = 12.65). Similar differences were observed for IN (*F*(3,404) = 4.94, *p* < 0.001, *η*^2^ = 0.03), PN (F(3,404) = 5.86, *p* < 0.001, *η*^2^ = 0.04), and MP (*F*(3,404) = 6.41, *p* < 0.001, *η*^2^ = 0.04), while EN did not differ (*F*(3,404) = 1.98, *p* = 0.110). Overall, smoking status related more consistently to nutrition attitudes than to knowledge.

**Table 9:** One-way ANOVA results for NKS and ASHN (total and subscales) by smoking status.

Variables	Never (*n* = 204) *M* ± SD	<10/day (*n* = 57) *M* ± SD	≥11/day (*n* = 91) *M* ± SD	Former (*n* = 56) *M* ± SD	*F*(3,404)	*p*	η^2^	Tukey pairs (*α* = 0.05)
NKS Total	47.93 ± 34.35	51.77 ± 26.01	54.39 ± 23.04	41.73 ± 22.44	3.47	0.010	0.02	≥11/day > Never, Former; <10/day > Former
ASHN Total	78.53 ± 10.86	75.00 ± 12.91	72.30 ± 12.65	78.37 ± 13.32	6.47	< 0.001	0.04	Never > < 10/day, ≥11/day; Former > ≥ 11/day
IN	21.43 ± 3.51	20.49 ± 3.71	19.50 ± 4.85	20.41 ± 4.83	4.94	< 0.001	0.03	Never > ≥ 11/day
EN	18.86 ± 4.49	19.24 ± 5.10	18.75 ± 4.97	20.50 ± 4.65	1.98	0.110	0.01	—
PN	18.08 ± 4.20	16.50 ± 4.47	16.13 ± 4.08	17.98 ± 3.83	5.86	< 0.001	0.04	Never > < 10/day, ≥11/day; Former > ≥ 11/day
MP	20.15 ± 3.80	18.75 ± 4.49	17.91 ± 4.65	19.48 ± 4.52	6.41	< 0.001	0.04	Never > < 10/day, ≥11/day; Former > ≥ 11/day

Values are mean ± SD. Smoking status categories are as reported by participants. NKS, Nutrition Knowledge Scale; ASHN, Attitudes Toward Healthy Nutrition; IN, Information on Nutrition; EN, Emotion for Nutrition; PN, Positive Nutrition; MP, Malnutrition. Two-tailed tests; *p* < 0.05 considered significant.

As shown in Table 10, age was positively associated with ASHN Total (*r* = 0.27, *p* < 0.001) and with the EN (*r* = 0.31), PN (*r* = 0.19), and MP (*r* = 0.23) subscales, indicating small-to-moderate increases in healthier attitudes with greater age. By contrast, no association was observed between age and NKS Total (*r* = −0.07, ns) or IN (*r* = 0.01, ns).

**Table 10:** Correlations of age with nutrition knowledge and healthy-eating attitudes (*N* = 408).

Variable	NKS Total	ASHN Total	IN	EN	PN	MP
Age	−0.07	0.27**	0.01	0.31**	0.19**	0.23**

Values are Pearson correlations. NKS, Nutrition Knowledge Scale; ASHN, Attitude Scale for Healthy Nutrition; IN, Information on Nutrition; EN, Emotion for Nutrition; PN, Positive Nutrition; MP, Malnutrition. **p < 0.01; *p < 0.05.

As shown in Table 11, higher NKS scores were modestly associated with lower healthy-eating attitudes overall (ASHN Total: *r* = −0.18, *p* < 0.001). Negative associations were also observed for IN (*r* = −0.21, *p* < 0.001), PN (*r* = −0.11, *p* = 0.020), and MP (*r* = −0.17, *p* < 0.001), whereas EN showed no association (*r* = −0.01, *p* = 0.760). Collectively, these small effects suggest that greater nutrition knowledge does not necessarily translate into more favorable attitudes across all domains, indicating a possible knowledge–attitude gap.

**Table 11:** Correlations between nutrition knowledge (NKS) and healthy-eating attitudes (*N* = 408).

Predictor	ASHN Total	IN	EN	PN	MP
NKS Total	−0.18**	−0.21**	−0.01	−0.11*	−0.17**

Values are Pearson correlations. NKS, Nutrition Knowledge Scale; ASHN, Attitude Scale for Healthy Nutrition; IN, Information on Nutrition; EN, Emotion for Nutrition; PN, Positive Nutrition; MP, Malnutrition. **p < 0.01; *p < 0.05.

**Figure 1:**
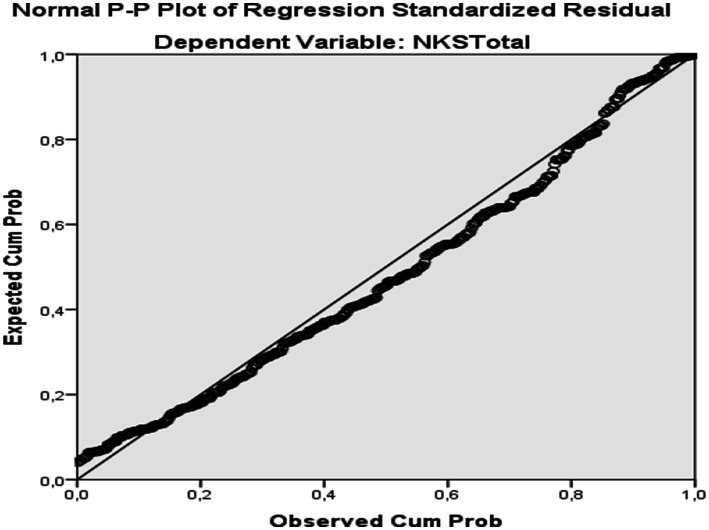
Normal P–P plot of regression standardized residuals for the model with NKS total as the outcome.

As shown in Table 12, a simultaneous multiple linear regression examined whether ASHN subscales were independently associated with NKS Total. The overall model was significant but explained a small proportion of variance (*R^2^* = 0.063; Adj. *R^2^* = 0.054). Controlling for the other subscales, IN (*β* = −0.17, *p* = 0.001) and MP (*β* = −0.16, *p* = 0.006) were negative predictors of NKS, whereas EN (*β* = 0.06, *p* = 0.270) and PN (*β* = 0.02, *p* = 0.620) were not significant. Diagnostics indicated no multicollinearity concerns (Tolerance = 0.64–0.79; VIF = 1.25–1.55) and approximately independent errors (*DW* ≈ 2.00). Residual-versus-fitted plots indicated approximate linearity and homoscedasticity, Cook’s distance and leverage did not identify influential cases, and the Normal P–P plot showed standardized residuals closely approximating normality (Figure 1). Higher scores on the IN and MP attitude facets were associated with lower nutrition-knowledge scores after adjustment, whereas affective and positive-nutrition attitudes (EN, PN) were unrelated to knowledge. Overall, ASHN subscales accounted for approximately 5–6% of variability in NKS, indicating that nutrition-related attitudes and nutrition knowledge were only weakly linked in this sample.

**Table 12:** Multiple linear regression examining associations between NKS total and ASHN subscales (IN, EN, PN, MP).

Predictor	*B*	SE B	*β*	*t*	*p*	Tolerance	VIF
Constant	80.72	7.91	—	10.20	< 0.001	—	—
IN	−1.06	0.32	−0.17	−3.29	0.001	0.79	1.25
EN	0.31	0.28	0.06	1.10	0.270	0.79	1.25
PN	0.16	0.33	0.02	0.48	0.620	0.68	1.45
MP	−0.95	0.34	−0.16	−2.74	0.006	0.64	1.55

Model fit: *R* = 0.252; *R*^2^ = 0.063; Adjusted *R*^2^ = 0.054; Durbin–Watson = 2.001; *F*(4, 403) = 6.815, *p* < 0.001.

NKS, Nutrition Knowledge Scale; ASHN, Attitude Scale for Healthy Nutrition; IN, Information on Nutrition; EN, Emotion for Nutrition; PN, Positive Nutrition; MP, Malnutrition.

## Discussion

This study clarified how adults’ NKS relates to their ASHN and how these constructs vary across physical activity, smoking, alcohol use, family chronic illness, and sociodemographic factors. Correlations between NKS and ASHN indices were small and frequently negative (e.g., ASHN Total *r* ≈ −0.18; IN *r* ≈ −0.21; PN *r* ≈ −0.11; MP *r* ≈ −0.17), and multiple regression showed small negative coefficients for IN and MP when predicting NKS after mutual adjustment. This pattern reflects construct distinctness rather than measurement failure: NKS captures declarative facts, whereas ASHN reflects evaluative orientation agreement with positive nutrition practices (PN/IN) and lower endorsement of malnutrition-congruent statements (MP). People with higher knowledge may also be more risk-attentive and thus more likely to recognize pitfalls that map onto MP items, producing modest inverse links. In some subgroups most visibly heavier smokers greater exposure to health information may not translate into more favorable attitudes, suggesting cognitive–affective misalignment or reactance (7). Unmeasured variables such as time scarcity, cost salience, diet restraint, self-regulation, or food environment plausibly shape knowledge acquisition and attitudes in different ways, further attenuating alignment in cross-section. The modest explained variance and marginal associations are consistent with two complementary explanations: measurement error in brief self-report indicators and construct mismatch, because declarative knowledge does not necessarily translate into motivation, self-regulation, or environmental opportunity. In future work, incorporating psychosocial determinants (e.g., autonomous motivation, self-efficacy, planning, habit strength) and contextual measures (e.g., time pressure, affordability, food availability) alongside richer behavioral indicators (diet quality indices and device-based activity) may improve model fit and clarify pathways.

Attitudes clustered with lifestyle, whereas knowledge did not. ASHN Total and its cognitively evaluative facets (IN and PN) increased monotonically from sedentary to high physical-activity categories, yet NKS did not vary by activity. Parallel gradients appeared for smoking and alcohol: never- and former-smokers and non-drinkers showed more favorable attitudes (higher PN, lower MP), while NKS was comparable across alcohol groups and paradoxically higher among heavier smokers. These converging gradients are consistent with behavioral clustering (23–25): individuals oriented toward one health domain (activity, non-smoking) also endorse more favorable nutrition attitudes, likely via shared determinants such as self-efficacy, planning, goal setting, and health identity (16, 26, 27, 41). Knowledge, by contrast, may accumulate through schooling, media, or clinical encounters and thus remains orthogonal to day-to-day lifestyle patterns (42). Notably, EN was the least discriminating subdomain across activity and lifestyle strata, whereas IN and PN differentiated groups clearly; interventions that emphasize beliefs and concrete practices may therefore tap into the same self-regulatory capacities that sustain physical activity and non-smoking, while purely affective appeals may be less sensitive to existing lifestyle orientation (16, 17).

Sociodemographic gradients favored attitudes over knowledge. Educational attainment displayed a robust stepwise association with ASHN (Master’s > Bachelor’s/Associate > High school) without a corresponding gradient in NKS, consistent with the idea that formal education shapes values, norms, and executive capacities that support favorable appraisals of healthy eating (43, 44) more than it augments domain-specific facts beyond a general threshold. Married participants showed more favorable ASHN, plausibly reflecting shared household routines, meal planning, and diffusion of health priorities within families (10, 29, 30). Age correlated positively with EN, PN, and MP, but not with NKS, suggesting that life-course prioritization of health may gradually strengthen attitudes even when factual knowledge remains relatively stable (11, 13, 45). Taken together, these gradients support a view in which attitudes are embedded in roles and contexts, whereas knowledge is comparatively context-insensitive once basic literacy is achieved (12).

Within a planned-behavior perspective, IN/PN align more closely with attitudinal and control beliefs that feed intention and action than NKS does (20, 46, 47). Dual-process accounts similarly predict that affective dispositions (EN) and cognitive evaluations (IN/PN) need not rise with knowledge unless interventions also engage motivation, identity, and habit (14, 15). Durable improvements likely require pairing informational content with self-regulatory tools (goal setting, implementation intentions, self-monitoring, environmental prompts), motivational interviewing or values-consistent framing, and social scaffolding (family/peer support) (18, 19, 22, 48). The paradoxical combination of relatively higher NKS and less favorable ASHN among heavier smokers exemplifies where alignment strategies clarifying goals, addressing ambivalence, building coping plans, and translating knowledge into feasible micro-behaviors should supersede additional didactic content (49, 50).

### Limitations

The cross-sectional design precludes causal inference; reverse or bidirectional pathways are plausible (e.g., favorable ASHN may promote physical activity, which further reinforces attitudes). Physical activity was assessed using a single self-reported item; therefore, misclassification due to recall or social desirability cannot be ruled out. Convenience sampling limits generalizability and invites selection biases. All variables were self-reported; the single-item physical-activity measure captures frequency but not intensity or domain and likely carries more error than validated multi-item instruments or devices. Although NKS and ASHN showed acceptable psychometrics, common-method variance, item directionality (reverse-scored items), and content mismatch between facts and attitudes may attenuate associations. We did not adjust for potentially important covariates (e.g., BMI, socioeconomic status, diet quality, food insecurity, food environment), any of which could confound knowledge attitude links or lifestyle gradients. The number of comparisons was substantial; future work should consider multiplicity control or pre-registration. The temporal and cultural context may shape both attitudes and behaviors; replication in diverse settings and time periods is warranted.

This study pairs validated measures of knowledge and attitudes with rich lifestyle and sociodemographic data, yielding a clear account of knowledge attitude discordance and attitude behavior clustering. Standardized effect-size reporting improves transparency. Because attitudes more than knowledge track with physical activity and other lifestyle factors, findings support shifting intervention content toward motivation, self-regulation, and environmental supports. Future work should use prospective/experimental designs to test attitude-focused components beyond education (e.g., implementation intentions, self-monitoring, habit formation), incorporate device-based activity measures and fuller dietary/contextual assessments, and conduct mediation (self-efficacy, autonomous motivation) and moderation (education, marital status, smoking) analyses. Attention to food environments, time pressure, and price sensitivity will be critical for converting gains in ASHN into sustained dietary improvements.

The use of convenience sampling limits the generalizability of these findings. In addition, the sample’s gender composition was skewed toward male participants relative to distributions typically reported for comparable populations, which may have affected the magnitude and stability of some estimates. To better evaluate sensitivity to sample composition, future studies should apply post-stratification weights (e.g., by sex, age, and education) and re-estimate key models as robustness checks. Accordingly, the present results should be interpreted as descriptive for this sample rather than as population-level prevalence estimates.

## Conclusion

In this adult sample, nutrition knowledge and healthy-eating attitudes were only weakly aligned, sometimes showing small negative associations. Attitudes—but not knowledge—varied systematically across physical activity and lifestyle factors, and age and education related more consistently to attitudes than to knowledge; heavier smoking also illustrated knowledge–attitude misalignment. These patterns suggest that information alone is unlikely to produce sustained change without motivation- and self-regulation–focused supports and reductions in contextual barriers. Accordingly, practice and policy may benefit from embedding attitude-focused components within broader health-behavior platforms, particularly physical-activity promotion and tobacco-control programs. In clinical care, brief screening for attitudinal barriers and readiness can guide counselling beyond information provision (e.g., motivational interviewing and implementation intention planning), while policy implementation through primary care, community programs, and workplace wellness may enable scalable, resource-efficient gains.

## Data Availability

The raw data supporting the conclusions of this article will be made available by the authors, without undue reservation.
